# Assessing the impact of land surface temperature on urban net primary productivity increment based on geographically weighted regression model

**DOI:** 10.1038/s41598-021-01757-7

**Published:** 2021-11-15

**Authors:** Xue-Yuan Lu, Xu Chen, Xue-Li Zhao, Dan-Jv Lv, Yan Zhang

**Affiliations:** 1 College of Big Data and Intelligent Engineering, Southwest Forestry University, Kunming, 650000 China; 2 College of Forestry, Southwest Forestry University, Kunming, 650000 China; 3 College of Mathematics and Physics, Southwest Forestry University, Kunming, 650000 China

**Keywords:** Environmental impact, Environmental health

## Abstract

Urbanization had a huge impact on the regional ecosystem net primary productivity (NPP). Although the urban heat island (UHI) caused by urbanization has been found to have a certain promoting effect on urban vegetation NPP, the factors on the impact still are not identified. In this study, the impact of urbanization on NPP was divided into direct impact (NPP_dir_) and indirect impact (NPP_ind_), taking Kunming city as a case study area. Then, the spatial heterogeneity impact of land surface temperature (LST) on NPP_ind_ was analyzed based on the geographically weighted regression (GWR) model. The results indicated that NPP, LST, NPP_dir_ and NPP_ind_ in 2001, 2009 and 2018 had significant spatial autocorrelation in Kunming based on spatial analytical model. LST had a positive impact on NPP_ind_ in the central area of Kunming. The positively correlation areas of LST on NPP_ind_ increased by 4.56%, and the NPP_ind_ caused by the UHI effect increased by an average of 4.423 gC m^−2^ from 2009 to 2018. GWR model can reveal significant spatial heterogeneity in the impacts of LST on NPP_ind_. Overall, our findings indicated that LST has a certain role in promoting urban NPP.

## Introduction

Urbanization, as one of the most extreme anthropogenic land-use/land-cover (LULC) changes, strongly affects the terrestrial ecosystem carbon cycle^1–3^. Urbanization is reflected in the replacement of vegetation areas which can directly alter the regional terrestrial ecosystem^4^. Furthermore, the habitat of urban vegetation is also severely affected by urbanization^5^, and a typical example is urban heat island (UHI) effect. Vegetation net primary productivity (NPP), the net amount of dry organic biomass accumulated by plants per unit area and time^6,7^, reflects the changes in the structure and operation of the terrestrial ecosystem^8,9^. As an important component of the terrestrial ecosystem carbon cycle, NPP quantifies the growth of vegetation, which is related to the amount of vegetation and the growth environment in an area^10,11^. Therefore, NPP is a common indicator to assess urbanization process impacts on urban vegetation^12,13^.

Urbanization has two opposite impacts on vegetation depending on different urban development stages^14–18^. Rapid urban expansion leads to a dramatic reduction in natural vegetation and cropland, which is a direct impact^14^, especially appear in the newly expanded urban areas. For the direct impact, the unprecedented urbanization in China in recent decades have led to drastic land-cover change^19^, which can undoubtedly reduce regional NPP^20,21^. For instance, intensive urbanization in Shenzhen City resulted in an 80% decrease in NPP^22^. Liu et al*.* found that approximately 309.95 GgC was lost over 13 years in Wuhan City, which was mainly due to the conversion from cropland to built-up areas^23^. On the contrary, urbanization may also increase NPP mainly inside the old urban area through UHI effect, artificial irrigation or by introducing highly productive plants and reducing the impact of climate factors^15–18^, which is an indirect impact. For the indirect impact, it was first discovered in Tokyo that the higher photosynthetic rate of the street trees observed in the central district of city^24^. In China, it was found that the vegetation growth of most cities is clearly improved by the indirect impact, which offsets about 40% of the direct NPP loss caused by the direct impact^2^. In addition, Guan et al*.* based on multi-source remote sensing data to establish and analyze both the long-term direct and indirect impacts of urbanization on NPP^17^. The results indicated that the urbanization has also resulted in an apparently positive indirect impact on NPP and the urban temperature was the main driving force of the indirect impact. However, a systematic understanding of the driving mechanism of indirect impact of NPP is largely unknown. Urban areas tend to have higher temperatures than the surroundings, referred to as urban heat island (UHI), largely resulted from the rise of impervious surface^25–27^. And UHI as an important factor of global warming, has not been studied deeply how to drive the growth of urban vegetation. As a major parameter associated with surface radiation and energy exchange, Land surface temperature (LST) is able to modulate the air temperature of the layer immediately above the earth surface^28–30^. And previous UHI-related articles have reviewed and found that 44% of these manuscripts used LST as a synonym for UHI when examining urban thermal environments^31,32^. Consequently, LST retrieved from infrared remote sensing imagery has been widely applied to study the phenomenon of urban heat island effect^33,34^. So, LST can be used as the UHI indicator to study the promotion of UHI on indirect impact of NPP in this study.

Although the previous studies have put forward some valuable insights into the forces driving of urban NPP changes, including the increase and decrease of NPP^2,22–24^. The research on spatial heterogeneity still needs to be further studied. Urban areas are characterized by complex land cover types with highly heterogeneous sizes and development states^35^. A limited number of studies have considered the spatial patterns of urbanization impacts. They divided cities into different buffer zones according to urban expansion intensities^14,17^. However, the main method used in these studies is the traditional ordinary least square (OLS) regression model. The OLS model tend to ignore the role of the spatial location of the different observations required^36^. This leads to biased estimations, which might not provide a reliable basis for understanding the impact of UHI on the indirect impact in a city. But, the geographically weighted regression (GWR) model, which allows the estimated parameters to accommodate potential spatial differences varying across region^37,38^, is more conducive to study the influence of LST on the indirect impact of NPP for different areas of the city area.

Kunming, as the capital of Yunnan province, one of the largest plateau mountain cities in southwestern China^17^ has undergone rapid urbanization over the last few decades, with an expanding urban area and population^37^. Overall, in this study, we choose Kunming as a case study area, the main objectives are: (1) To clarify the urbanization process, the UHI effect, NPP expansion of the city and the distribution of direct and indirect impact of NPP using spatial autocorrelation analysis. (2) To provide an estimation of the indirect impact of urbanization on NPP and a certain understanding of the driving mechanism of UHI on NPP through revealing the spatial distribution of correlation coefficients of LST on indirect impact of NPP based on the OLS and GWR models.

## Results

### The spatial features of NPP, IS and LST

**Figure 1:**
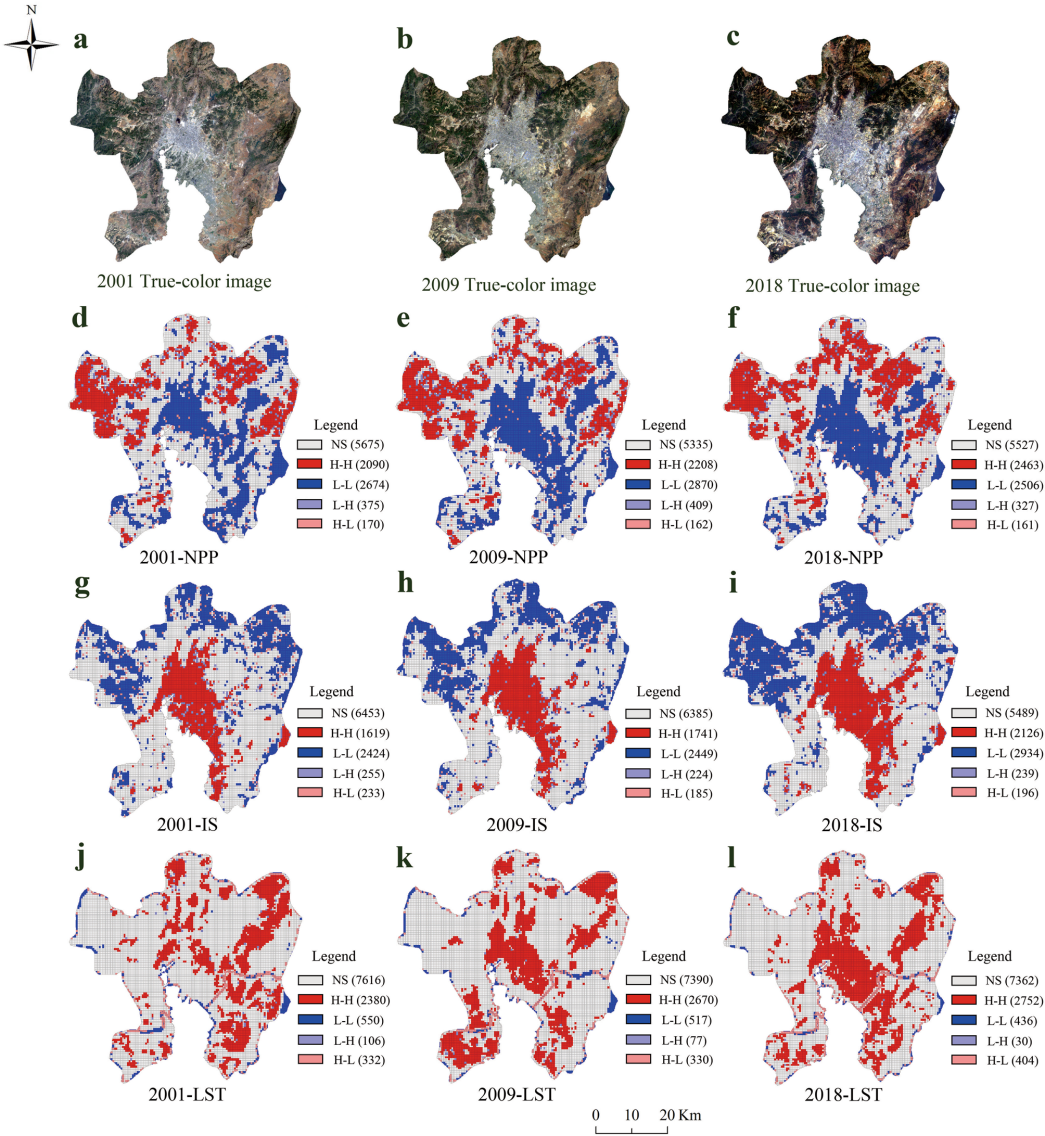
LISA cluster maps of NPP, IS and LST. (**a**–**c**) are the true color images of Kunming; (**d**–**l**) are the LISA cluster maps of NPP, IS and LST in 2001, 2009, and 2018. NS means not significant. H–H means high–high clusters. L–L means low–low clusters. L–H means low–high clusters. H–L means high–low clusters. In addition, NPP is net primary productivity. IS is impervious surface. LST is land surface temperature.

In order to assess the spatial agglomeration, global Moran’s indexes of NPP, land surface temperature (LST) and impervious surface (IS) were calculated using Geoda software^39^. As shown in Table 1, the global Moran’s I of NPP, IS and LST were greater than 0 (*p* < 0.001), meaning that they are positive spatial autocorrelation in Kunming. In other words, NPP, LST and IS among the city areas tend to gather in space. However, the global Moran’s I only shows whether there is agglomeration in the city, but it does not reveal in which area the agglomeration occurs. To further understand the evolution in local spatial distribution, the local indicators of spatial association (LISA) map of NPP, IS and LST was calculated (Fig. 1).

**Table 1:** The global Moran’s I of NPP, IS and LST in 2001, 2009, and 2018. GMI denotes the globe Moran’s index. *Z* is the standardized value which denotes the *Z*-Scores. *P* is the significance test level value which denotes the *P*-values. NPP is net primary productivity. IS is impervious surface. LST is land surface temperature.

Years		GMI	*Z*	*P*
2001	NPP	0.521	103.597	0.001
	IS	0.572	116.944	0.001
	LST	0.283	58.459	0.001
2009	NPP	0.519	104.104	0.001
	IS	0.595	118.292	0.001
	LST	0.275	56.306	0.001
2018	NPP	0.503	105.414	0.001
	IS	0.593	118.852	0.001
	LST	0.252	50.301	0.001

The local Moran LISA maps (Fig. 1d–f) revealed that the NPP mostly were concentrated in the LL (low-low) and HH (high-high) clusters areas. Specifically, LL clusters of NPP distributed mainly inside the city and the Chenggong district, a new investment and development zone. From 2001 to 2018, the LL clusters of NPP gradually expanded to Chenggong and airport area. Interestingly, a shrinking trend of the LL clusters was observed in 2018, which had no more concentrated areas than in 2009, especially in Chenggong district. Because of the intensification of urban development, the value of regional NPP is similar to the surrounding grid points and shows insignificant characteristics. The clusters of NPP distributed mainly in north, northwest and northeast regions, where more vegetation zones. Correspondingly, the clusters of NPP could be an index for the urbanization. HH clusters indicated less vegetation inside the urban area, while LL clusters indicated more vegetation in the forest (Fig. 1a–c). In addition, HL (high-low) clusters appeared in or around the LL clusters, representing the existence of vegetation in the city. HH clusters of IS (Fig. 1g–i) mainly gathered in the city area corresponding to true color image (Fig. 1a–c). While the distribution of LL clusters of IS was approximately consistent with the HH clusters of NPP. LST is geographically concentrated in HH clusters areas (Fig. 1j–l). And HH clusters of LST is mainly concentrated in the central, northeast and south of Kunming. The HH clusters of LST gradually expanded with the development of city through time.

In general, the HH clusters of IS, LL clusters of NPP and the HH clusters of LST were roughly distributed in city area of Kunming. The urban expansion of Kunming can be obtained from the HH clusters of IS (Fig. 1g–i). From 2001 to 2018, the urban area gradually expanded to the airport, Chenggong district and the vicinity of Dianchi Lake. At the same time, urbanization has led to the decrease of vegetation in urban newly expansion areas, resulting in a decrease in NPP. NPP correspondingly showed LL clusters in urban areas. As the intensity of urbanization increases, the city’s surface has become “urban heat”. As a result, LST produces HH clusters within the city areas. In summary, the urbanization process would bring the reduction of NPP and the strengthening of the UHI effect. The HH clusters of LST and LL clusters of NPP have similar distributions in the central of Kunming especially in 2009 and 2018 which provides a basis for studying the impact of UHI on urban NPP.

### The spatial features of direct and indirect impact of NPP

The direct impact of NPP caused by the reduction of vegetation coverage is defined as NPP_dir_. And the indirect impact of NPP result from anthropogenic and climatic factors is defined as NPP_ind_. As shown in Table 2, the global Moran’s I of NPP_dir_ were observed to be 0.152 and 0.271 in 2009 and 2018 respectively, and the global Moran’s I of NPP_ind_ were 0.347 and 0.378 in 2009 and 2018 respectively, indicating a significant positive spatial autocorrelation in NPP_dir_ and NPP_ind_ (*p* < 0.001).

**Table 2:** The global Moran’s I of NPP_dir_ and NPP_ind_ in 2001, 2009, and 2018. NPP_dir_ is the direct impact of urbanization on NPP and NPP_ind_ is the indirect impact of NPP.

Years		GMI	*Z*	*P*
2009	NPP_dir_	0.152	31.197	0.001
	NPP_ind_	0.348	70.725	0.001
2018	NPP_dir_	0.271	56.021	0.001
	NPP_ind_	0.378	74.398	0.001

**Figure 2:**
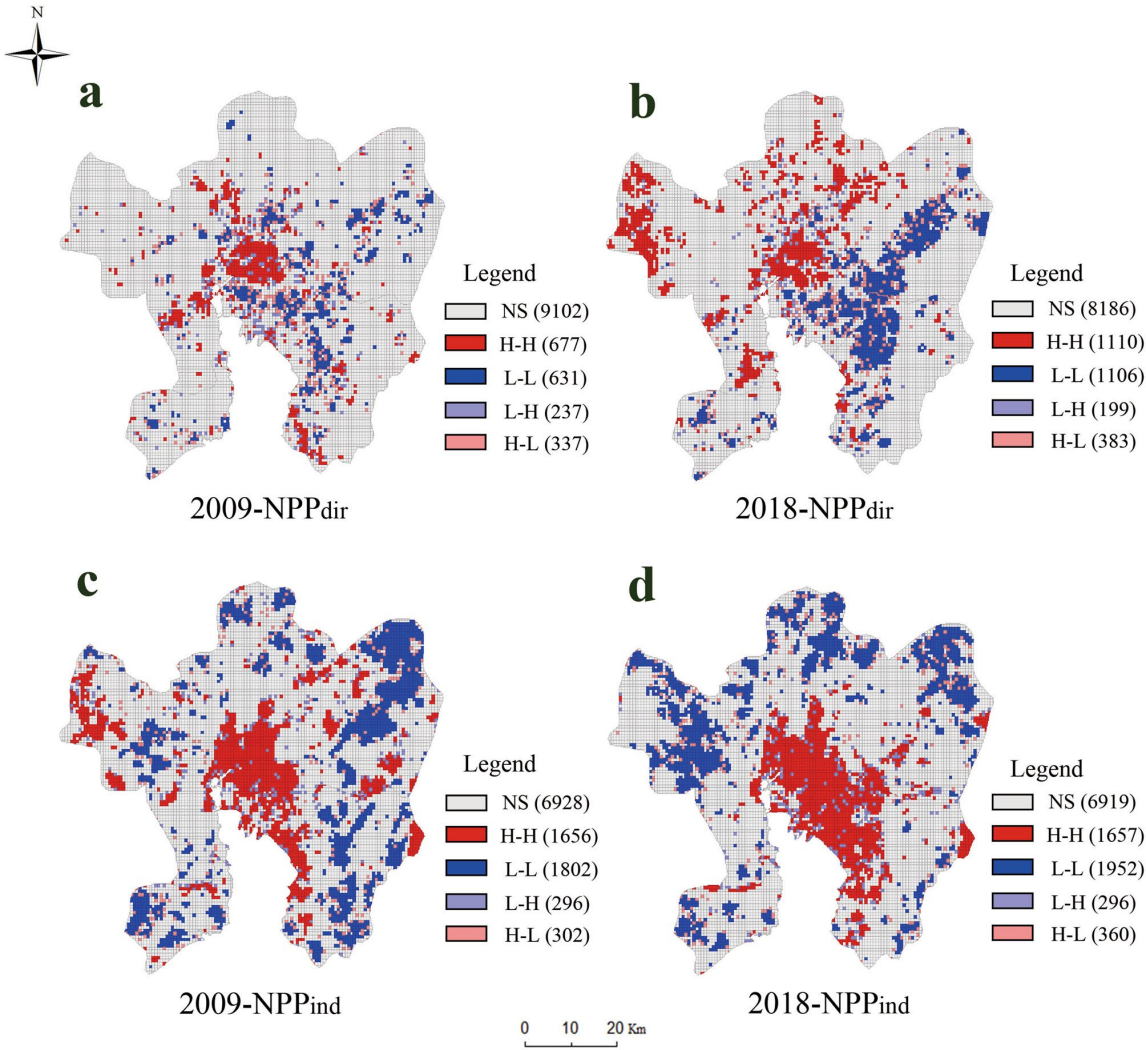
LISA cluster maps of NPP_dir_ (**a**,**b**) and NPP_ind_ (**c**,**d**) in 2009 and 2018. NPP_dir_ is the direct impact of urbanization on NPP and NPP_ind_ is the indirect impact of NPP.

The LISA cluster map of NPP_dir_ and NPP_ind_ (Fig. 2) further illustrates the local spatial distribution geographically. The NPP_dir_ mostly concentrated in the LL and HH clusters. LL clusters mainly concentrated in the newly expanded urban areas such as Chenggong district, representing the most obvious area of direct impact of NPP. From 2009 to 2018, the area of the LL clusters has expanded significantly and is consistent with the HH clusters of IS (Fig. 1), which proves that newly expanded urban areas mainly reflect the NPP_dir_ because of the transformation of LULC. On the contrary, with the increase of urban intensification, NPP_ind_ expanded from the central area to the surrounding area in the city. The indirect impact of NPP was mainly reflected in the better growth of urban vegetation within city, so the HH clusters of indirect impacts mostly are concentrated in urban areas. Corresponding to LISA cluster map of LST in 2009 and 2018, the HH clusters of LST and NPP_ind_ have roughly similar distribution ranges.

### Regression analyses of OLS model

LST is negatively correlated at the 1% significance level (Table 3), indicating that UHI effect will lead to decreased indirect impact of NPP on the entire city area of Kunming. But it is inconsistent with our argument that UHI effect will promote the growth of urban vegetation. Because the traditional OLS regression method assumes that there are no heterogeneous differences in Kunming city, and estimates the “global” parameters of the explanatory variables. Specifically, the forest areas in the north, northwest and northeast of Kunming are also included, but the UHI effect in these areas is not obvious and may not promote vegetation growth, which lead to negative correlation between LST and NPP_ind_ in the whole area. From 2009 to 2018, the correlation coefficient increased from − 6.019 to − 2.994. The negative correlation of LST on NPP_ind_ became smaller in the whole areas.

**Table 3:** Global regression analyses of LST on NPP_ind_ (OLS model). * Indicates a statistically significant *p*-value (*p* < 0.01).

Years	Coefficient	Std. error	*t* value	Pr. (> |*t*|)
2009	− 6.019*	0.103	− 58.580	0.000
2018	− 2.994*	0.069	− 43.909	0.000

### Regression analyses of GWR model

We applied the GWR method to explore the spatial heterogeneity of the LST on NPP_ind_ of Kunming. A summary of estimated coefficients is given in Table 4. The standard residual of coefficient estimation between − 2.5 and 2.5 is considered as the high reliability^37^. Most of the standard residual of coefficient estimates in the study area are reliable with the proportion is 98.8% and 97.9% in 2009 and 2018 respectively (Table 4).

**Table 4:** Descriptive statistics of regression coefficients in the GWR model.

Years	Min	Max	Mean	Std. err.	− 2.5 < Std.Res < 2.5(%)
2009	− 27.487	8.971	− 7.703	0.608	98.770%
2018	− 9.075	10.742	− 3.112	0.399	97.940%

**Figure 3:**
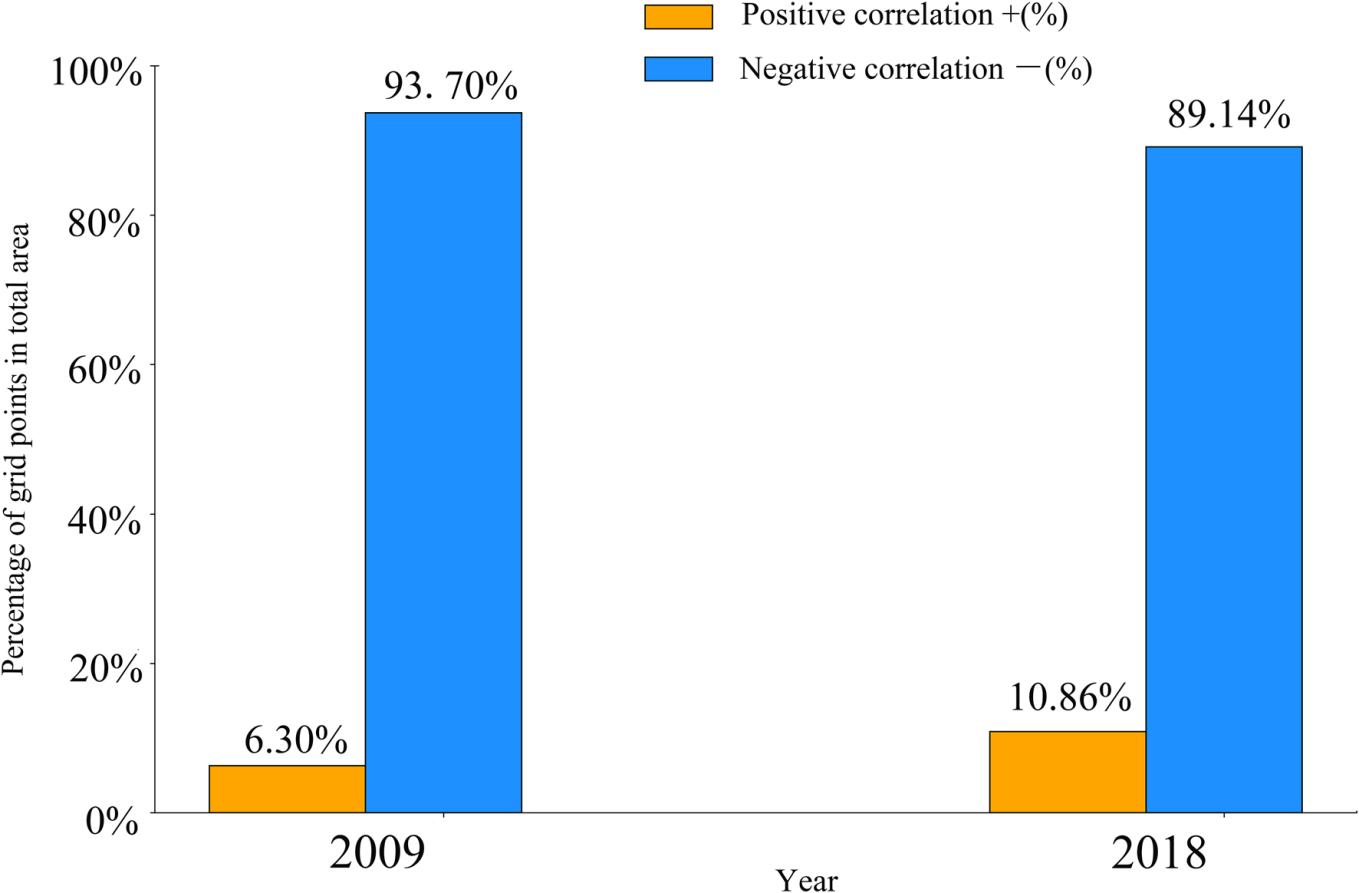
Percentage of positive and negative correlation of LST on NPP_ind_ in Kunming city. The orange column represents percentage of positive correlation area of LST on NPP_ind_ in total area. The blue column represents percentage of positive correlation area of LST on NPP_ind_ in total area.

**Figure 4:**
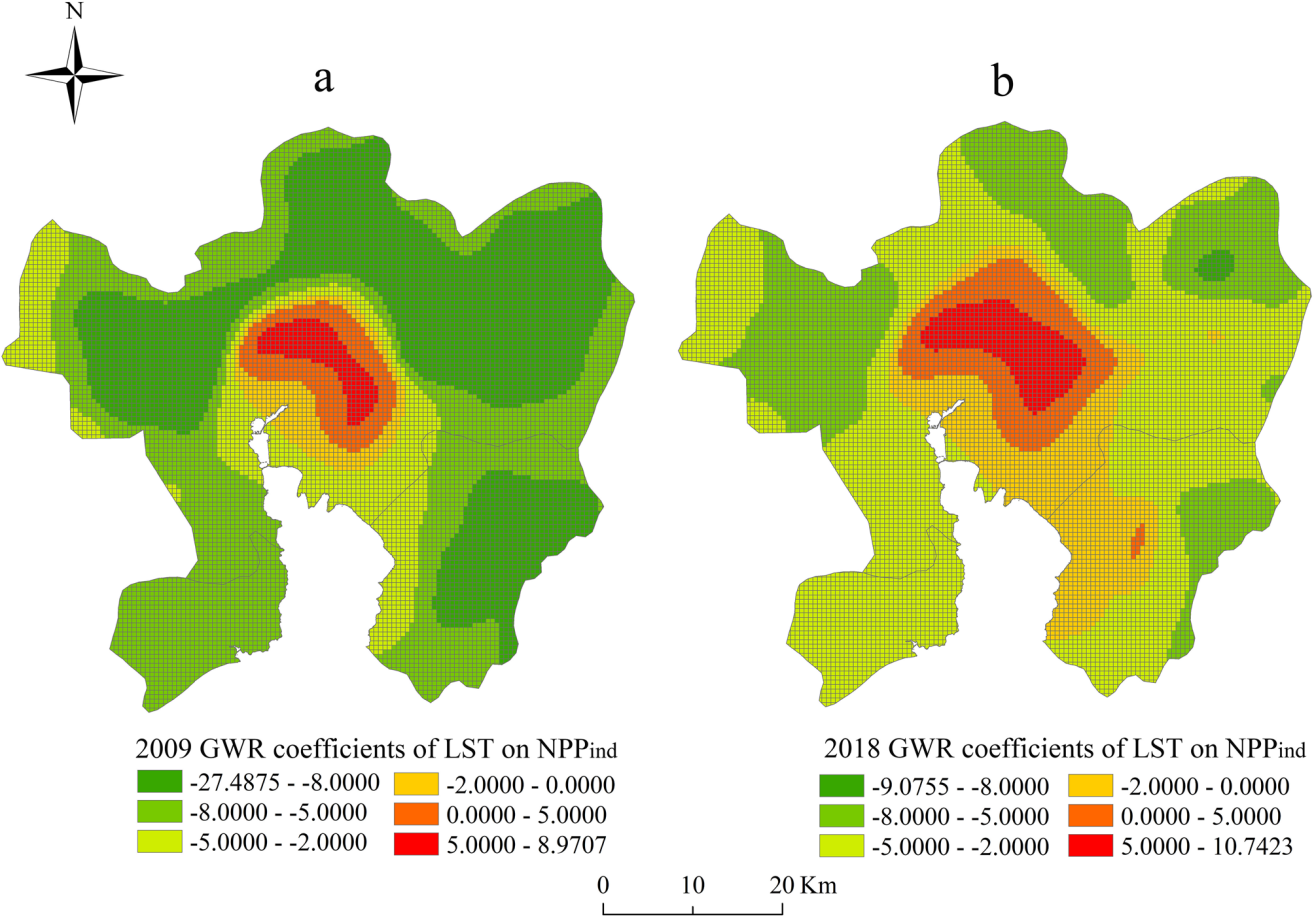
The spatial distribution of the GWR local coefficients of LST on NPP_ind_.

As displayed in Fig. 4, the estimated coefficients of the LST on NPP_ind_ range from − 27.488 to 8.971, and from − 9.076 to 10.742 in 2009 and 2018. Respectively, implying that the impact of LST varies greatly from region to region. The positive coefficients are mainly concentrated in the central city shown in red and orange in Fig. 4, which is consistent with the areas of HH clusters of LST that characterizes UHI effect. The high temperature caused by the UHI effect has positively impact on increasing the urban vegetation NPP of the central city area in a certain extent. However, low estimated coefficients were observed in the surrounding area probably due to lower LST. From the perspective of spatial changes, the areas with positive coefficients gradually expanded from the main city area to the surrounding area from 2009 to 2018. A small part of the southeast of Kunming, the interior of Chenggong district, also showed a positive correlation until 2018. As shown in Fig. 3, the positive correlation area of the study area increased from 6.30 to 10.86% from 2009 to 2018. The rapid urbanization has led to an increase in the scope of LST, which can be seen in Fig. 1. In addition, due to the promotion of LST on NPP, NPP_ind_ increased by an average of 4.423 gC m^−2^ from 2009 to 2018 in Kunming (Table 5).

**Table 5:** Descriptive statistics of NPP_ind_ in Kunming. NPP_ind_ is the indirect impact of NPP. Grid count is the number of grid points where NPP_ind_ > 0. The unit of max, min and mean is gC m^−2^ year^−1^.

Years	Grid count (NPP_ind_ > 0)	Min	Max	Mean
2009	243	0.297	204.729	14.415
2018	403	0.125	139.873	18.838

### Comparison of OLS and GWR

In order to prove whether the GWR model is better than the OLS model for the results in this study, we compared the R^2^ and AIC statistics respectively in the GWR and OLS model. The GWR model revealed a higher R^2^ (0.496 and 0.486 in 2009 and 2018 respectively) than that of the OLS model (0.143 and 0.388 in 2009 and 2018 respectively), indicating that the GWR model is better fitted. Then we compared the AIC statistics. When the AIC value differs by more than 3, the lower the AIC value, the better the fit^40^. As shown in Table 6, the AIC value for the GWR was approximately 4314 less than the AIC value of OLS in 2009, and 5312 less in 2018. To sum up, the GWR model is better fitted than the OLS model. The analysis of the estimated coefficients of the explanatory variables in the previous section also shows that the wider the range of variable coefficients, the greater the spatial variability of the contribution and influence of LST on NPP_ind_, which illustrates the appropriateness of the GWR model in providing a better explanation for the local estimation.

**Table 6:** Comparison of AIC values between OLS and GWR models.

Years		OLS	GWR
2009	Adjusted R^2^	0.143	0.496
	AIC	134,948.273	130,634.719
2018	Adjusted R^2^	0.388	0.486
	AIC	128,760.397	123,448.120

## Discussion

The urbanization has led to dramatic carbon flux fluctuations. Most of the previous studies focused on the reduction of NPP caused by urban expansion^18–21^. A typical example is that Wen et al*.*^41^ quantified the large urban expansions occurred in most provinces of China and were accompanied with huge NPP losses. They have only considered the total NPP when study about the driving factors of NPP. If the indirect impact was not considered, we could only make the conclusion that the influencing factors brought by urbanization have reduced the urban NPP. But the NPP loss just consider land-cover replacement is inaccurate, because compensation effect of climate and artificial factors will reduce the NPP loss. As shown in Fig. 1d–f, the LL clusters of NPP in cities have expanded with urban development which was in good agreement with the results of previous studies. In fact, the reduced NPP was the result of the area of vegetation being replaced mainly in newly expanded areas of city, but the remaining vegetation was much more productive after the urbanization. This phenomenon has been discovered in early studies. For example, Gregg et al*.*^42^ reported that enhanced biomass of vegetation in New York City compared with that of rural sites. Takagi and Gyokusen^24^ also found that the higher photosynthetic rate in the urban core due to the air pollutant. They all began to realize the urban vegetation had much more productive affected by urbanization, in essence, this is due to the indirect impact of urbanization on urban vegetation NPP. Then there are some studies to quantify this indirect impact. And the indirect impact was found to be able to offset the direct loss of vegetation NPP caused by replacing vegetated surfaces in the urban area^16,17^. In this study, the HH clusters of NPP_ind_ has expanded significantly with the urbanization from the perspective of spatial distribution (Fig. 2). It showed that NPP_ind_ in Kunming mainly existed in urban areas, especially in the old city, and it gradually expanded to the Chenggong district. The mean value of NPP_ind_ increased by 4.423 gC m^−2^ from 2009 to 2018 (Table 5). This result is similar to that of Guan et al*.*^17^ that the indirect impact can promote NPP more in the old city region. In addition, separating the indirect impact of NPP can contribute to identify the real relationship between UHI effect and NPP. The total NPP of vegetation cannot represent the growth status of urban vegetation. Because of the direct impact, even though the vegetation growth is promoted by UHI effect, urban NPP will decrease with urbanization. All these conclusions could only be drawn by separating indirect impacts of urbanization on NPP. Thus, it is necessary to separate the indirect impact of NPP, in order to figure out the real relationship between UHI effect and urban vegetation NPP.

The driving factors of NPP have been examined through previous research^14,17^. Due to the complexity of urban spatial heterogeneity, the coefficients of different factors varied by land use and region under different urbanization intensities. In order to divide the areas with different urbanization intensity, Tian et al*.*^14^ divided Beijing into four eco-regions including the Capital Core Functional Region (CCFR), the Capital Extended Functional Region (CEFR), the New Developing Functional Region (NDFR), and the Ecological Reservation and Developing Functional Region (ERDFR). Guan et al*.*^17^ divided Kunming into old city (OC), expansion area (EA), sub-urban area (SA) and non-urban area (NA). They have taken into account the spatial patterns of the urbanization impact by dividing the buffer zone, and did regression analysis by region to analyze the indirect impact of climate factors on NPP. However, these studies rely on traditional regression models, and have failed to reveal the spatial changes in each buffer zone. The regional linear regression will result in cliff-jumping changes in the boundaries between regions and also make the relationship between different regions unable to be expressed. Compared with the regression analysis, GWR can accurately display the regional variation of coefficient^36^. As the results of the impact of LST on NPP_ind_, the results from the GWR (Fig. 4) revealed more specific information which cannot be revealed by OLS model (Table 3). GWR divided Kunming into 10,984 fishnet grids with 500 × 500 m resolution to obtain the regression coefficients of each grid locally taking into account the spatial weight matrix. Since GWR considers the spatial information of each point, it has higher accuracy compared to sub-regional regression. The spatial distribution of regional correlation coefficients revealed the specific regions where LST promoted NPP_ind_. Pei et al*.*^20^ indicated that the increases of NPP might be correlated with the effects of UHI caused by urban land development around some regions that experienced rapid urbanization, as well as the arid regions in northwest China. In this study, the NPP_ind_ also found to be positive correlated with LST in the city of Kunming, where has experienced rapid urbanization in recent years. Guan et al*.*^17^ also mentioned that climate condition in the old city was years ahead of that in the expansion area, as a result of higher temperature in the old city. In Fig. 4, the vegetation in the old city also has more productivity affected by the UHI effect. Compared with the previous studies^18,20^, GWR specifically clarify the areas of LST promotes NPP_ind_ over time rather than dividing urbanization intensity regions artificially. Overall, GWR has reference significance for analyzing the driving factors of NPP in urban areas with spatial heterogeneity.

The major uncertainties of this study mainly come from CASA model and other factors that may affect NPP. First, although the CASA model is widely used for NPP inversion^4,11,17,43^, the data processes and the parameter value definition could bring some bias to the NPP results^17^, which also influencing NPP_dir_ and NPP_ind_. As an input data of CASA model, the monthly total solar radiation obtained by Kriging interpolation may have error caused by inadequate meteorological data sites. Subsequent research can consider other improved models to overcome the limitation of fewer sites^44,45^. And as an important static parameter of CASA model, the maximum light use efficiency ε_max_ has been shown to be different among different vegetation types, ecosystems and seasons. But the ε_max_ values in this study may be more suitable for the whole country, which adopted from the study of Zhu et al*.*^46^. So further adjustment of ε_max_ for urban vegetations is necessary. Then, due to the lack of field measurement of NPP data in Kunming, we used the MODIS NPP product (MOD17A3) to assess the accuracy of the NPP from CASA model. The mean estimation values of CASA and the MODIS product were extracted from 500 random points except the districts in the central city, because the NPP on building land was not estimated in the MODIS product^11^. The validation results of these points indicating that the CASA model is suitable for estimating the NPP in Kunming city (*R* = 0.73, *P* < 0.01). Secondly, this study separated the impact of urbanization on NPP into NPP_ind_ and NPP_dir_, so it would be meaningful to consider other influencing factors of NPP. For the total NPP, at regional scale, variation of precipitation was found to dictate most of the inter-annual variation of NPP of the tropical region. And in the mid northern latitudes the variation of NPP seems to be attributed to the relative variability and mean of temperature^47^. So Kunming NPP may be more related to temperature. Therefore, this study provides a certain understanding of the driving mechanism of LST on NPP_ind_ in urban areas. However, other factors that may affect the NPP, such as the urban dry island, urban rain island and urban CO_2_ concentration should also be considered in subsequent studies^20,22,48^. The NPP was due to the vegetation replacement in the process of urbanization, but the changes of the type, form and maintenance of the vegetation should also be explored in further study. For example, among the vegetation in different urban structures, the vegetation in old urban areas is older and have longer growth times, which is more adaptable to the urban environment. While vegetation in new urban areas is mostly newly transplanted trees with younger age. In addition, this study divided the study area into 500 × 500 m grids, so it would be meaningful to divide into finer grids. And how to increase the time point and expand the scope of study area to 32 major cities in China are also interesting directions for future work, which will be of great significance in advancing our knowledge of urbanization and terrestrial ecosystems. In general, although some conclusions were obtained in this study, we believe that the conclusions obtained in this study are credible and valuable. But further efforts are still required for an in-depth exploration of the indirect impact of urbanization on NPP.

## Conclusion

Urbanization has a huge influence on regional NPP. In this study, first, the direct and indirect impact of urbanization on NPP were separated. Then spatial variations of the correlation coefficient between LST and NPP_ind_ were analyzed both globally and locally with the support of OLS and GWR models. The main conclusions can be summarized as follows:

1. From 2001 to 2018, the results of spatial autocorrelation analysis showed that the study area has experienced an accelerated urbanization process. Most of the urban sprawl was concentrated in Chenggong district and the airport area of northeast of Kunming. With the expansion of the city and the decrease of vegetation coverage, NPP has shown a corresponding decreasing trend and LST is higher in the urban areas.
2. After dividing the impact of urbanization on NPP into NPP_ind_ and NPP_dir_, the spatial autocorrelation analysis showed that NPP_ind_ and NPP_dir_ differ in the spatial agglomeration area. In the process of urbanization, the scope of LL clusters of NPP_dir_ were mainly appeared in the newly expanded urban areas, which was due to the reduction of NPP caused by land cover replacement in the new urban areas. However, the HH clusters of NPP_ind_ were concentrated in the central area of Kunming city and gradually expanded to the surrounding areas with the urban development, which was similar to the area affected by higher LST.
3. Urbanization leads to the decrease of vegetation which caused the reduction of NPP in urban area. However, urbanization has also brought positive effects, promoting the growth of vegetation, such as UHI effect. In this study, we mainly studied the spatial heterogeneity of the impact of LST on NPP_ind_. Compared with the OLS model, the GWR model revealed that LST has a positive impact on NPP_ind_ in the central city of Kunming. And the positive correlation area expanded by 4.56% from 2009 to 2018. Particularly in recent 10 years, there also appeared positive correlation areas in Chenggong district. And the mean value of NPP_ind_ increased by 4.423 gC m^−2^ from 2009 to 2018. These results indicated that the urban LST which characterizing UHI effect can promote the growth of vegetation in the central city of Kunming.

## Materials and methods

### Study area

**Figure 5:**
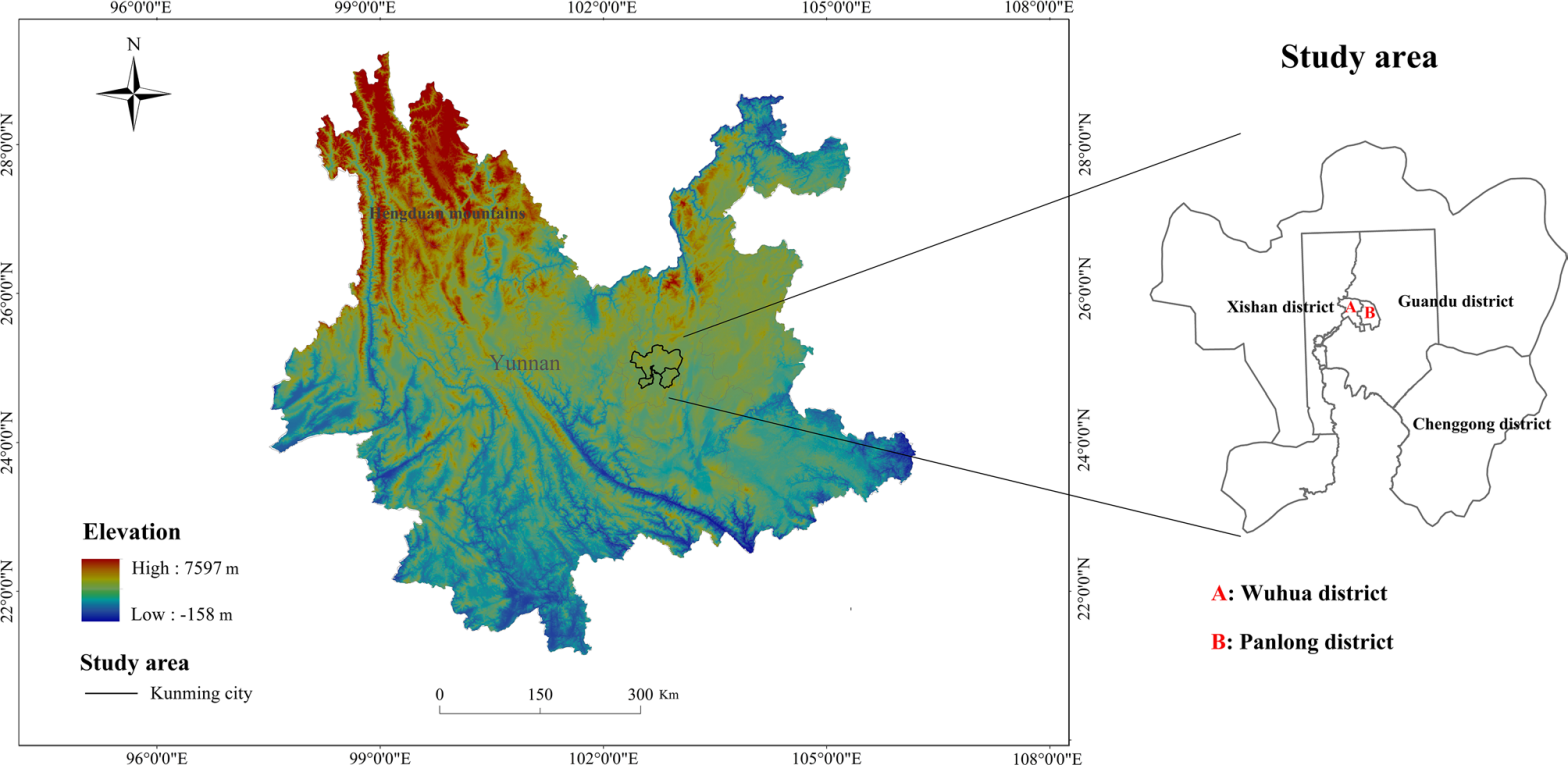
The location of the study area. This map was generated by Arcgis10.2 (https://www.esri.com/en-us/arcgis).

Yunnan Province, bordered by the Qinghai-Tibet Plateau in the north, and the Hengduan Mountains in the northwest, is a component of the Yunnan-Kweichow Plateau. Kunming city (24.13°N–25.18°N, 102.20°E–103.03°E), as the capital of Yunnan, was selected as the study area. Kunming city located at the northern of the Dianchi Basin in Yunnan, which is a representative of low-latitude plateau cities. As shown in Fig. 5, the city has a total area of 2602.46 km^2^, an average elevation of 1900 m, and is surrounded by mountains on three sides. Affected by the circulation of the southwest monsoon and the adjustment of the water surface of Dianchi Lake, a natural environment with spring seasons, abundant rainfall, long sunshine and perennial southwest wind has been formed^49^. Located in the northern subtropical monsoon climate zone, Kunming has typical temperate climate characteristics. However, the climate shows great seasonal heterogeneity, with humid summers and dry winters, and most of the precipitation occurs during the growing season (April to October). In order to distinguish the spatial patterns of the urbanization impacts, the total urban area was shown as the Wuhua district, Panlong district, Xishan district, Guandu district and Chenggong district (Fig. 5). The Wuhua district and Panlong district areas are the earliest developed old city in Kunming, which has the highest degree of impervious surface. Then Xishan District and Guandu District have a higher degree of urbanization. And the establishment of Chenggong New District in recent years has greatly promoted the rapid development of Kunming.

### Data source and preprocessing

The Landsat and moderate-resolution imaging spectroradiometer (MODIS) images used in this study were selected based on the least amount of cloud (cloud cover < 10%). Landsat Thematic Mapper (TM)/Operational Land Imager (OLI) were downloaded from the Geospatial Data Cloud (http://gscloud.cn/) and the United States geological survey (USGS) science center (http://glovis.usgs.gov/). The MODIS products were also obtained from the USGS (http://glovis.usgs.gov/). The monthly meteorological data used in this study include average temperature, total precipitation and the total solar radiation. The climate data, were derived from 29 climatological stations around Kunming city, which were provided by the China Meteorological Administration (http://data.cma.cn/). The digital elevation model (DEM) data was obtained from LPDAA of NASA (http://www.gdem.aster.ersdac.or.jp/), and the vector data was extracted from the GADM database (https://www.gadm.org/). All Landsat and MODIS images were rectified to the universal transverse mercator (UTM) projection and world geodetic system 1984 (WGS84) datum.

The LST was inverted by Landsat TM/OLI images based on the radiative transfer equation (RTE) method^50,51^ at a spatial resolution of 30 m in 2001, 2009 and 2018. Based on the linear spectral mixing analysis model (SMA)^52^, Landsat images were used to analyze the abundance of impervious surface in Kunming covering the same time point as LST. The percentage of impervious surface in a single pixel was used to quantify the intensity of urban expansion.

In order to meet the requirements of heterogeneity of small-scale urban vegetation study, we simulated the 30 m NPP in 2001, 2009 and 2018 based on the Carnegie Ames Stanford Approach (CASA) model^46^. In the NPP estimation framework, first, to obtain the normalized difference vegetation index (NDVI), the Landsat TM/OLI images were used to extract NDVI calculated by Landsat infrared (IR) band and near-infrared (NIR) band. MCD12Q1 at spatial resolution of 500 m covering the study area closer to the observation time of Landsat images were selected as land cover classification data. To control the CASA model, the land cover types data from MCD12Q1 were further reclassified into different vegetation types, including evergreen broad-leaved forest (EBF), deciduous broad-leaved forest (DBF), evergreen needle-leaved forest (ENF), deciduous needle-leaved forest (DNF), mixed forest (MF), shrub, urban land, cropland, grass-land, unused land and water area. Then the cover classification data were resampled to 30 m. Because it is difficult to acquire meteorological data including monthly average temperature, total precipitation and the total solar radiation due to the limited number of meteorological stations, the meteorological data were used to interpolated into 30 m raster images using Kriging method^53^. Kriging method is the most widely used interpolation method. It thinks that any attribute of spatial continuously change is stochastic, and semi-variation function is used to analyze data^43^. The root-mean-square standardized of Kriging is close to 1, indicating that the standard error is accurate. In this study, the root-mean-square standardized of monthly average temperature, total precipitation and the total solar radiation are 1.8525, 0.7344 and 0.9671 respectively. The mean standardized of Kriging is close to 0, indicating that the results are unbiased. And mean standardized of monthly average temperature, total precipitation and the total solar radiation are − 0.0653, − 0.0510 and − 0.0636 respectively. These Kriging statistical errors illustrate the reliability of the interpolation results. Finally, according to the study of Zhu et al.^46^, the static parameters such as the maximum light use efficiency *ε*_max_ (gC MJ^−1^)^54^ were obtained.

### The direct and indirect impact of urbanization on NPP

In order to obtain the direct and indirect impact of urbanization on NPP, a specific method for separating and analyzing NPP_dir_ and NPP_ind_ was detailed as follows^17^. First, assuming that every pixel has ideal full vegetation coverage before urbanization which defined as $NPP_{{{\text{fv}}}}$. And assume that $NPP_{{{\text{fv}}}}$ does not change over time. Then, according to SMA model, the urbanization intensity (β) is represented by the percentage of IS of every pixel. $NPP_{{\text{h}}}$ is hypothetical NPP assuming that there is no indirect impact during the urbanization period, which is determined by the percentage of the non-urban surface (1 − β) and the $NPP_{{{\text{fv}}}}$ together, and are described in detail as the following:

1 $$NPP_{{\text{h}}} = \left( {1 - \beta } \right) \times NPP_{{{\text{fv}}}}$$

Thus, based on this concept (1), under ideal conditions, the hypothetical $NPP_{{\text{h}}}$ after urbanization in time t can be expressed as:

2 $$NPP_{{\text{h}}} \left( {x,\;t} \right) = \left[ {1 - \beta \left( {x,\;t} \right)} \right] \times NPP_{{{\text{fv}}}} \left( {x,\;t} \right)$$

where $NPP_{{\text{h}}}$(x, t_1_) is the NPP in pixel x at time t. And it is the hypothetical NPP value after urbanization, just considering the direct impact of land-cover changes; $\beta \left( {x,\;t} \right)$ is the urban expansion intensity; and $NPP_{{{\text{fv}}}}$(x, t) is the NPP value in pixel x at time t when it has full vegetation cover. The hypothetical $NPP_{{\text{h}}}$ in time t_0_ can be expressed as:

3 $$NPP_{{\text{h}}} \left( {x,\;t_{0} } \right) = \left[ {1 - \beta \left( {x,\;t_{0} } \right)} \right] \times NPP_{{{\text{fv}}}} \left( {x,\;t_{0} } \right)$$

And the hypothetical $NPP_{{\text{h}}}$ after urbanization in time t_1_ can be expressed as:

4 $$NPP_{{\text{h}}} \left( {x,\;t_{1} } \right) = \left[ {1 - \beta \left( {x,\;t_{1} } \right)} \right] \times NPP_{{{\text{fv}}}} \left( {x,\;t_{1} } \right)$$

Then the change in NPP due to urbanization from t_0_ to t_1_ is formula (4)–(3):

5 $$NPP_{{\text{h}}} \left( {x,\;t_{1} } \right) - NPP_{{\text{h}}} \left( {x,\;t_{0} } \right) = \left[ {1 - \beta \left( {x,\;t_{1} } \right)} \right] \times NPP_{{{\text{fv}}}} \left( {x,\;t_{1} } \right) - \left[ {1 - \beta \left( {x,\;t_{0} } \right)} \right] \times NPP_{{{\text{fv}}}} \left( {x,\;t_{0} } \right)$$

Since the ideal full vegetation cover pixel $NPP_{{{\text{fv}}}}$ does not change over time:

6 $$NPP_{{{\text{fv}}}} \left( {x,\;t_{0} } \right) = NPP_{{{\text{fv}}}} \left( {x,\;t_{1} } \right)$$

Then, we can calculate the direct impact at time t_1_ as follows:

7 $$NPP_{{{\text{dir}}}} \left( {x,\;t_{1} } \right) = NPP_{{\text{h}}} \left( {x,\;t_{1} } \right) - NPP\left( {x,\;t_{0} } \right) = \left[ {\beta \left( {x,\;t_{0} } \right) - \beta \left( {x,\;t_{1} } \right)} \right] \times NPP_{{{\text{fv}}}} \left( {x,\;t_{0} } \right)$$

where $NPP_{{{\text{dir}}}}$(x, t_1_) is the direct impact NPP in pixel x at time t_1_. The difference between $NPP_{{\text{h}}}$(x, t_1_) and the NPP at t_1_ should be the indirect impact of urbanization on NPP. The indirect impact at time t_1_ as follows:

8 $$NPP_{{{\text{ind}}}} \left( {x,\;t_{1} } \right)= NPP\left( {x,\;t_{1} } \right) - NPP_{{\text{h}}} \left( {x,\;t_{1} } \right)$$

where $NPP_{{{\text{ind}}}}$(x, t_1_) is the indirect impact NPP in pixel x at time t_1_. As $NPP\left( {x,\;t_{1} } \right)$ is the NPP value estimated from CASA model after urbanization in pixel x at time t_1_, bring formula (3) into formula (8) to get the indirect effect NPP:

9 $$NPP_{{{\text{ind}}}} \left( {x,\;t_{1} } \right) = NPP\left( {x,\;t_{1} } \right) - \left[ {1 - \beta \left( {x,\;t_{1} } \right)} \right] \times NPP_{{{\text{fv}}}} \left( {x,\;t_{0} } \right)$$

According to the formula (9), we could calculate the direct and indirect impacts of urbanization on NPP from 2001 to 2009 and 2001 to 2018.

### Spatial autocorrelation analysis

Spatial autocorrelation analysis is used to determine whether there is spatial clustering of NPP, LST, IS, NPP_dir_ and NPP_ind_ of Kunming in 2001, 2009 and 2018. In order to meet the calculation limits of Geoda in this study, the IS, LST, NPP, NPP_dir_ and NPP_ind_ at a spatial resolution of 30 m of Kunming city were resampled to 500 × 500 m fishnet grid. Then, using Geoda software^39^ to obtain the global Moran’s index and local Moran’s index of the above indicators. While, global Moran’s I reflects the global spatial autocorrelation between different geographical regions and local Moran’s I reflects the local similarities and differences between neighboring areas^55^. The two variables of the z-score and the p-value respectively refer to the spatial correlation level between neighboring areas and its corresponding significance level^35^.

(1) Global spatial autocorrelation

Global spatial autocorrelation is used to measure the spatial autocorrelation. This study used the global Moran's I and its statistical test to analysis the spatial dependence of NPP, LST, IS, NPP_dir_ and NPP_ind_. It was first proposed in 1950 by the Australian statistician Parker Moran^56^. The global Moran's I statistics of spatial autocorrelation can be expressed as:

10 $$I = \frac{{\mathop \sum \nolimits_{i = 1}^{n} \mathop \sum \nolimits_{j = 1}^{n} W_{ij} \left( {Y_{i} - \overline{Y}} \right)\left( {Y_{j} - \overline{Y}} \right)}}{{S^{2} \mathop \sum \nolimits_{i = 1}^{n} \mathop \sum \nolimits_{j = 1}^{n} W_{ij} }}$$

11 $$S^{2} = \frac{1}{n}\mathop \sum \limits_{i = 1}^{n} \left( {Y_{i} - \overline{Y}} \right)^{2} ,\quad \overline{Y} = \frac{1}{n}\mathop \sum \limits_{i = 1}^{n} Y_{i} ,$$

where $n$ is the total number of observation area. As $Y_{i}$ is the Observation value in the *i*-th region. $W_{ij}$ is the spatial weight matrix, j = 1, 2, …, *n*. *n* is the number of cross-sectional observation units. The global Moran's I values between − 1 and 1, if the Moran’s I is greater than 0, it means that this attribute value has a positive correlation in the study area. In contrast, if the Moran’s I is less than 0, it means that the attribute value has a negative correlation. The larger the value, the greater the degree of spatial autocorrelation.

If the calculated global Moran’s index shows that this attribute value has spatial autocorrelation, the Z value can be used to test its significance. Z value calculation formula is as follows:

12 $$Z = \frac{I - E\left( I \right)}{{\sqrt {Var\left( I \right)} }},\quad E\left( I \right) = \frac{ - 1}{{n - 1}},\quad Var\left( I \right) = \frac{{n^{2} W_{1} - nW_{2} + 3W_{0}^{2} }}{{W_{0}^{2} \left( {n^{2} - 1} \right)}}E^{2} \left( I \right),$$

Z value can be used to test the spatial correlation. When Z > 0 and significant, there have spatial autocorrelation. When Z = 0, there have random distribution. When Z < 0 and significant, there have negative spatial correlation.

(2) Local spatial autocorrelation

Compared with global spatial autocorrelation, local spatial autocorrelation is used to calculate the spatial correlation degree between each spatial object and its neighboring objects in a region. The calculation of local Moran’s I is similar to the global Moran’s I. Anselin proposed the definition of the local Moran’s I in 1995^57^. The calculation of local Moran’s I is shown as follow:

13 $$I_{i} = \frac{{\left( {Y_{i} - \overline{Y}} \right)}}{{S^{2} }}\mathop \sum \limits_{j = 1}^{n} W_{ij} \left( {Y_{j} - \overline{Y}} \right)$$

14 $$S^{2} = \frac{1}{n}\mathop \sum \limits_{i = 1}^{n} \left( {Y_{i} - \overline{Y}} \right)^{2} ,\quad \overline{Y} = \frac{1}{n}\mathop \sum \limits_{i = 1}^{n} Y_{i} ,$$

The definitions of *n*, $Y_{i}$, $W_{ij}$ and n are the same as the global Moran’s index in the previous section (i = 1, 2, …, *n*, j = 1, 2, …, *n*).

The LISA cluster map can reflect where the indicators are clustered in the space. Local Moran’s I and LISA cluster map can be divided into four space-related patterns, namely high–high cluster (HH), low–low cluster (LL), low–high cluster (LH), high–low cluster (HL). HH clusters indicates that the high-value region is surrounded by other high-value regions. LL clusters represents a low-value region is surrounded by other low-value regions. Similarly, LH clusters indicates that the low-value region is surrounded by other high-value regions, and HL clusters represents that a high-value region is surrounded by other low-value regions^36,55^.

### Geographically weighted regression model

The geographically weighted regression model (GWR) can be used to examine the spatial heterogeneity of the effect of LST on NPP_ind_. The geographically weighted regression model (GWR) is a local spatial technique that can be used to examine the spatial variabilities of regression parameters and model performance. Compared with the ordinary least squires (OLS) model, the GWR model carries out separate regressions at each location, considering only other observations within a specific distance to that location^35^. The model can be expressed as follow^37^:

15 $$y_{i} = \beta_{0} \left( {u_{i} ,v_{i} } \right) + \mathop \sum \limits_{k} \beta_{k} \left( {u_{i} ,v_{i} } \right)x_{ij} + \varepsilon_{i}$$

where *y* is the dependent variable. *x* is the independent variable. *k* is the number of dependent variables. $\left( {u_{i} ,\;v_{i} } \right)$ represents the geographic coordinates of the *i*-th unit. $\beta_{0} \left( {u_{i} ,\;v_{i} } \right)$ is a constant term, $\beta_{k} \left( {u_{i} ,\;v_{i} } \right)$ is the local regression parameter to be estimated at the regression point $\left( {u_{i} ,\;v_{i} } \right)$ which represents the longitude and latitude coordinates of the *i*-th grid point. $\varepsilon_{i}$ is the random error term.

According to Tobler’s first law of geography, one thing is related to another, and more related to the nearer things than to the distant things^58^. Therefore, the GWR model adopting a distance decay function in cooperation with a weight matrix for calibration. The parameters estimation adopts weighted least square method:

16 $$\beta \left( {u_{i} ,v_{i} } \right) = \left( {X^{T} W\left( {u_{i} ,v_{i} } \right)X} \right)^{ - 1} \left( {X^{T} W\left( {u_{i} ,v_{i} } \right)Y} \right)$$

where $W\left( {u_{i} ,\;v_{i} } \right)$ is a diagonal weight matrix. With the change of $W\left( {u_{i} ,\;v_{i} } \right)$ and $\beta \left( {u_{i} ,\;v_{i} } \right)$. The choice of weighting is crucial in the GWR, as it defines how many neighbors should be included in the matrix. In this study, we selected the bi-square weighting function to calculate the spatial weight matrix. The adaptive bandwidth was adopted to determine the number of nearby observations. And the bandwidth is optimized according to the Akaike Information Criterion (AIC)^59^.
